# High accuracy of component positioning and restoration of lower limb alignment using robotic medial unicompartmental knee arthroplasty

**DOI:** 10.1002/ksa.12484

**Published:** 2024-10-06

**Authors:** Emanuele Diquattro, Jonathan Lettner, Marco Adriani, Robert Prill, Mikhail Salzmann, Roland Becker

**Affiliations:** ^1^ Orthopaedic‐Traumatology and Prosthetic Surgery and Revisions of Hip and Knee Implants IRCCS Istituto Ortopedico Rizzoli Bologna Italy; ^2^ Center of Orthopaedics and Traumatology University Hospital Brandenburg an der Havel Brandenburg Germany; ^3^ Department of Medical and Surgical Specialties, Radiological Sciences, and Public Health University of Brescia Brescia Italy; ^4^ Faculty of Health Sciences Potsdam Germany

**Keywords:** accuracy, ligament tension, MAKO, robotics, unicondylar knee arthroplasty

## Abstract

**Purpose:**

Unicondylar arthroplasty was performed using robotic medial unicompartmental knee arthroplasty (R‐mUKA) and gap‐balancing instrumentation. Our hypothesis was that robotic unicondylar knee arthroplasty accurately restores component positioning and lower limb alignment when compared to preoperative planning with actual implantation throughout the range of knee motion due to proper knee balancing.

**Methods:**

Data were collected prospectively and were analysed for patients undergoing R‐mUKA. A cemented UKA was implanted using the MAKO® robotic system. Lower limb alignment at 0°, 30°, 45°, 60° and 90° of flexion was recorded of the native knee, with the trial components in place and finally after component implantation. A spacer according to the femorotibial gap was introduced and the alignment was measured. The position of the final component was planned based on three‐dimensional computed tomography images before making the bone cuts. The positioning of the femoral and tibial components was analysed in all three planes.

**Results:**

A total of 52 patients were included (mean age 66.3 ± 6.7 years; 34 males, 18 females). The difference in femoral component position after planning and final implantation was 0.04° ± 0.58° more valgus in the coronal plane (*p* = 0.326) and 0.6° ± 1.4° more flexion relative to the sagittal plane (*p* = 0.034). The tibial component was placed in the coronal plane in 0.3° ± 0.8° of more varus (*p* = 0.113) and in the sagittal plane in 0.6° ± 1.2° of more posterior tibial slope (*p* = 0.001). Lower limb alignment of the native knee in extension was 5.8° ± 2.6° of varus and changed to 3° ± 2.1° varus after UKA (*p* ≤ 0.01).

**Conclusion:**

R‐mUKA helps to achieve the target of alignment and component position without any significant differences to the planning. Ligament balancing causes non‐significant changes in component position. It allows optimal component position even for off‐the‐shelf implants respecting the patient's specific anatomy.

**Level of Evidence:**

Level II.

**Abbreviations**
AP: anteroposterior
CT: computer tomography
ML: mediolateral
PCA: posterior condylar axis
ROM: range of motion
R‐UKA: robotic assisted unicondylar knee arthroplasty
sTEA: surgical transepicondylar axis
UKA: total knee arthroplasty

## INTRODUCTION

The success in unicondylar knee arthroplasty (UKA) relies on precise component position and effective soft tissue management. Alteration of the joint line orientation by more than 2 mm, changes in the posterior tibial slope by more than 2°, varus alignment greater than 3°, a posterior slope greater than 5° and a difference in coronal alignment of more than 6° between the femoral and tibial components are associated with increased failure rates after UKA [1, 9, 33].

Comparing conventional and robotic arm‐assisted unicondylar knee arthroplasty (R‐mUKA), higher accuracies have been demonstrated for both femoral and tibial component positions in favour of R‐mUKA [2, 23, 26, 27]. While there is evidence for improved accuracy in component position, there is no corresponding improvement in functional and clinical outcomes when compared to conventional surgery [17, 18, 25, 31, 37, 39].

Despite accurate component positioning, improved knee function is not automatically guaranteed: overstuffing and instability can increase the risk of inferior outcome and early revision [6, 14, 16]. The joint capsule and ligaments, which are sites of nociception, play a crucial role in achieving good knee function [15].

Although there are already a few papers in the literature considering the topic of accuracy in implant positioning, in the current study, the analysis of the lower limb alignment was included considering the entire range of motion. Good implant position does not mean automatically good knee function, but the alignment throughout the range of motion will provide inside information about the soft tissue envelope and, thus, better knee function can be expected [33].

Thus, it is important to consider the alignment of the lower limb throughout the range of motion. In addition to component position, this approach provides indirect information about ligament tension.

Our hypothesis was that robotic unicondylar knee arthroplasty accurately restores component positioning and lower limb alignment when compared to preoperative planning with actual implantation.

## MATERIALS AND METHODS

A prospective observational cohort study was conducted with a consecutive patient cohort, all of whom underwent R‐mUKA at a single centre, between January 2022 and March 2024. A cemented UKA design (Restoris MCK, Stryker) was used in all cases, and the MAKO semi‐active robotic‐arm‐assisted system (Mako Surgical Corp. [Stryker]) was used during surgery. The study was approved by the ethical committee of the state of Brandenburg (AS 113[bB]/2016).

Patients with varus osteoarthritis with or without previous partial or total medial meniscectomy were scheduled for medial UKA and were included in the study. Patients presenting with co‐morbidities such as hip or ankle pathologies, back pain, inflammatory arthropathies, neurologic disorders, functional incompetence of collateral ligaments or valgus deformity before surgery were excluded. A total of 52 knees were included in the study.

Full leg weight‐bearing anteroposterior (AP), lateral and merchant views were obtained for preoperative planning. Computer tomography (CT) scans of the entire lower limb, including the hip, knee and ankle, were performed. The axial images were exported for segmentation using robotic software, which then produced a specific three‐dimensional model of the patient's anatomy. The following three‐dimensional reference axes were calculated based on the bony landmarks from the preoperative CT scan: (1) femoral mechanical axis: the axis between the hip centre and the most distal point of the trochlear groove; (2) trans‐epicondylar surgical axis (TEA): the line connecting the tip of the lateral epicondyle and the sulcus of the medial epicondyle; (3) posterior condylar axis (PCA): the line tangent to the outlines of the posterior condyles; (4) tibial mechanical axis: the axis connecting the midpoint between the tibial spines with the midpoint between the most prominent point on the medial and lateral malleolus of the talus; and (5) AP tibial axis: the axis between the centre of the posterior condylar line (PCL) insertion and the medial third of the tibial tubercle.

All intraoperative references were recorded during the surgery using the robotic system. Lower limb alignment at 0°, 30°, 45°, 60°, 90° and maximum degree of flexion was recorded at the beginning of the surgery, repeated after the trial components were in place and with the final cemented implants prior wound closure.

The implant position during planning and after final cementing was analysed. The varus/valgus position in the coronal plane and flexion position in the sagittal plane of the femoral component were measured. The varus‐valgus position and posterior slope of the tibial component were also measured.

Surgical planning was carried out before surgery by the Medical Product Specialist of the implant company and sent for review to the surgeon. Final planning was performed during the surgery after anatomical landmarks were recorded and alignment throughout the range of knee motion was analysed. The robotic system is also controlled by a medical product specialist throughout the surgery.

All data are given as mean and standard deviation of the mean. The one‐sample and two‐sample paired T‐Test were used to compare the lower limb alignment before and after surgery and the component positions after planning and final implantation. A *p*‐value of less than 0.05 was considered statistically significant.

## RESULTS

A total of 52 patients (52 knees) with complete pre‐ and intraoperative data were analysed. The mean age of the study cohort at the time of intervention was 66.3 ± 7.7 years (min. 52, max. 85 years.). Of these, 34 (65.4%) were male and 18 (34.6%) were female.

The differences between the planned and the final femoral component position were 0.04° ± 0.6° more valgus in the coronal plane (*p* = 0.326) and 0.6° ± 1.4° more flexion in the sagittal plane (*p* = 0.034).

The tibial component was placed in the coronal plane in 0.3° ± 0.8° of more varus (*p* = 0.113) and in the sagittal plane in 0.6° ± 1.2° of more posterior tibial slope (*p* = 0.001) (Table 1).

**Table 1:** Difference between the planned and the final femoral and tibial component position in the coronal and sagittal plane after implantation of robotic UKA.

Femur component				
	Planned		Final	
	Mean (range)	SD	Mean (range)	SD
Coronal alignment (medial/lateral tilt)	−0.1° (−3° to 1,5°)	0.9°	0.1° (−2° to 2°)	0.9°
Sagittal alignment (flexion)	3.2° (−4° to 7°)	2.5°	3.7° (−2° to 8°)	2.5°
Tibial component				
Coronal alignment (medial slope)	1.7° (−2° to 3°)	1.0°	2.0° (0°–3°)	0.8°
Sagittal alignment (posterior slope)	4.8° (2°–7°)	1.4°	5.5° (2°–8,5°)	1.7°

Abbreviations: SD, standard deviation; UKA, unicondylar knee arthroplasty.

The distribution of each parameter was obtained from the planning phase and final position of the components after the implantation of robotic UKA (Figure 1).

Figure 1Distribution of planned (grey dots) and final (black dots) femoral and tibial component position in both the coronal and sagittal plane after implantation of robotic unicondylar knee arthroplasty.

**Figure:**
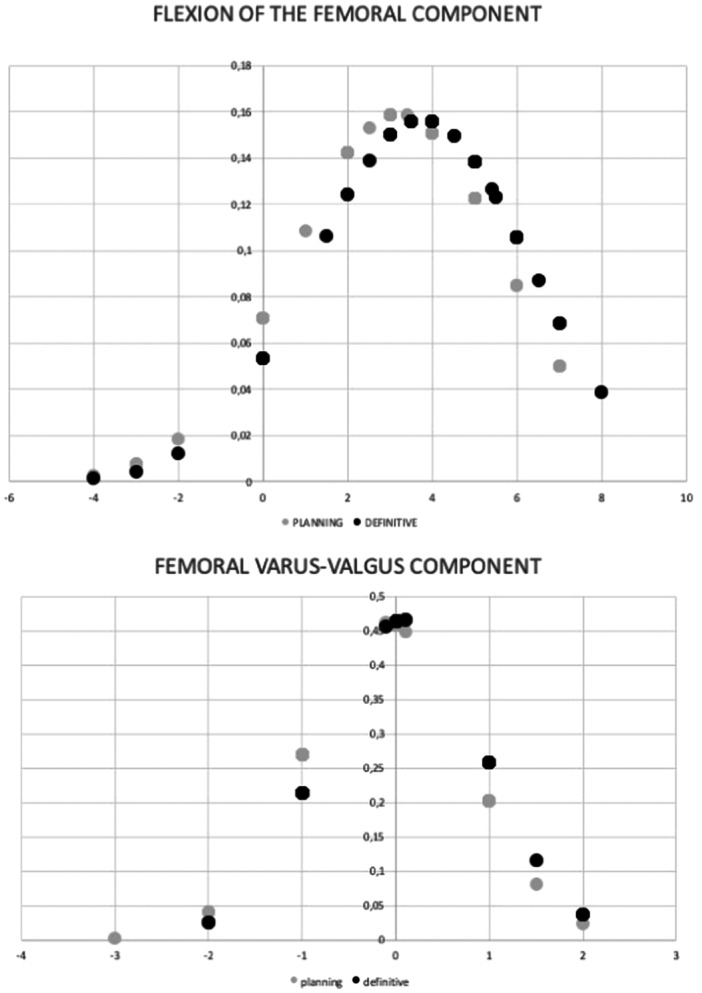

**Figure:**
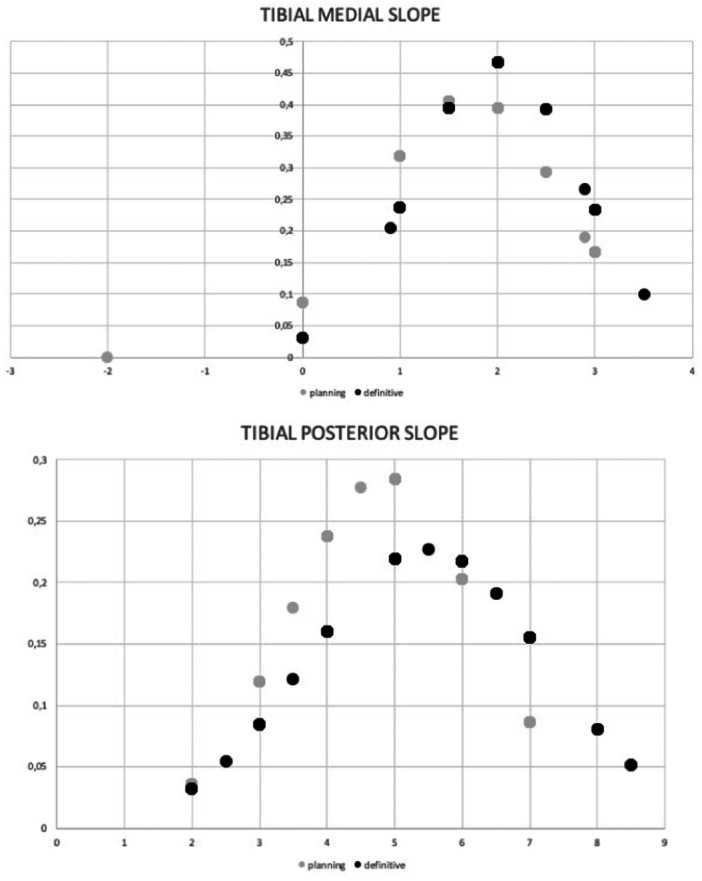

**Figure 2:**
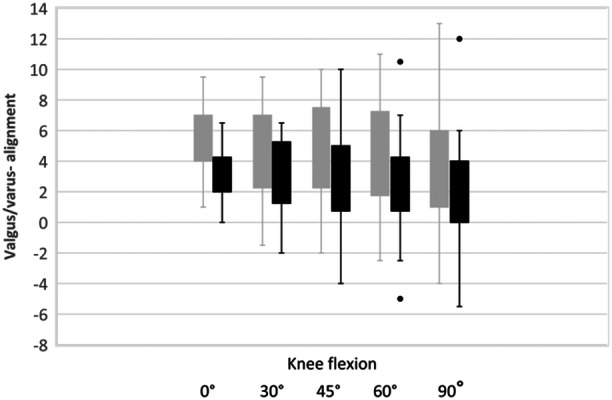
Mean and standard deviation of the alignment in the coronal plane in 0°, 30°, 45°, 60° and 90° of the native knee (grey columns) and after robotic arm‐assisted unicondylar knee arthroplasty (R‐mUKA) (black columns).

**Figure 3:**
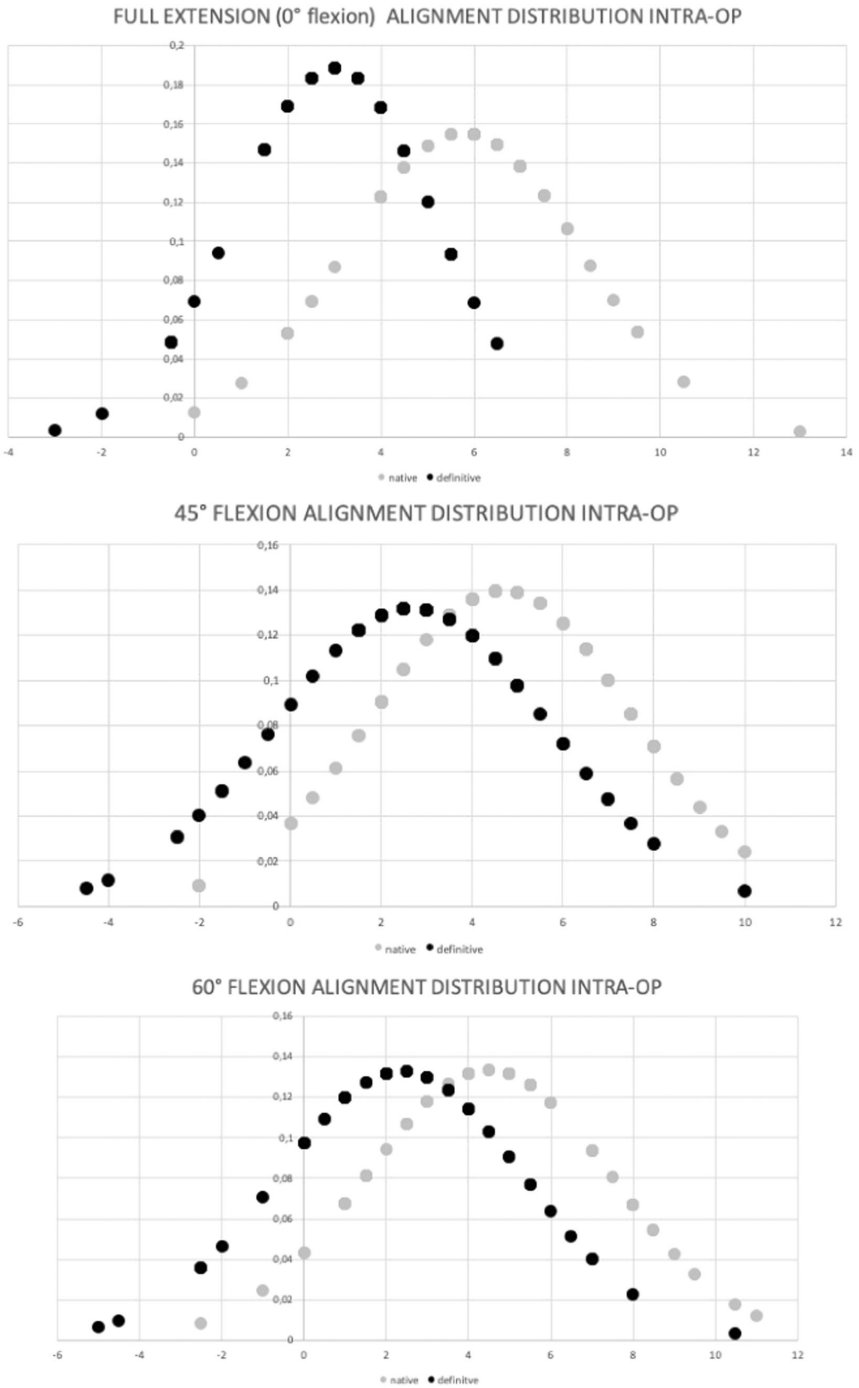
Alignment distribution of the native (grey dots) knee and after implantation (black dots) of the unicondylar knee arthroplasty (UKA) in 0°, 45° and 60° of knee flexion.

The lower limb alignment was recorded during surgery throughout the range of knee motion. The mean values were obtained from the native joint and finally with the cemented implants in place (Tables 2 and 3 and Figures 2 and 3).

**Table 2:** Paired sample test between the planned and the final component position in the coronal and sagittal plane after implantation of robotic UKA.

Component		T	*df*	One arm T‐Test	Two arm T‐Test
Pair 1 (sagittal plane)	Femur – planned Femur – final	−2,220	29	0.017	0.034
Pair 2 (coronal plane)	Femur – planned Femur – final	−1,000	29	0.163	0.326
Pair 3 (medial slope)	Tibial – planned Tibia – final	−1,635	29	0.056	0.113
Pair 4 (posterior slope)	Tibial – planned Tibial – final	−3,646	29	≤0.001	0.001

Abbreviation: UKA, unicondylar knee arthroplasty.

**Table 3:** Lower limb alignment in degrees of the native knee, after planning and after implantation of robotic UKA for the components at 0°, 30°, 45° 60°, 90° and maximal knee flexion.

	Native alignment	Planned alignment	Final alignment
0°	5.7° (SD 2.6°)	3.0° (SD 2°)	3.0° (SD 2.1°)
30°	4.5° (SD 2.8°)	3.0° (SD 2°)	3.0° (SD 2°)
45°	4.7° (SD 2.8°)	2.9° (SD 2.6°)	2.7° (SD 3°)
60°	4.5° (SD 3°)	2.4° (SD 2.6°)	2.0° (SD 3°)
90°	3.5° (SD 3.2°)	1.8° (SD 1.1°)	1.8° (SD 3°)
Max flexion	2.4° (SD 5.5°)	1.7° (SD 3.4°)	

Abbreviations: SD, standard deviation; UKA, unicondylar knee arthroplasty.

Furthermore, for the intraoperative alignment values obtained in full extension (0° of flexion), 30°, 45°, 60° and 90° of flexion, we calculated the mean values and standard deviations of the native measurements (pre‐cut) and those obtained with the definitive, and these were compared.

The standard deviation of the alignment in the coronal plane increased with flexion and was largest at 45°. The knees were left at a mean of 3° of varus, and none was overcorrected into valgus at full extension. A significant valgus alignment was noticed with an increase in flexion.

The distribution of the lower limb alignment is shown in 0°, 45° and 60°of knee flexion.

## DISCUSSION

The main finding was that R‐UKA accurately restores component positioning and lower limb alignment when compared to preoperative planning with actual implantation confirming the authors' hypothesis. The varus alignment was corrected on average by 2.7° compared to the pre‐surgery alignment, with particular respect to the soft tissue tension on the medial side of the knee. The soft tissue tension was respected by the gapping between the femoral and tibial components. No gapping is expected on the medial side in extension but up to 1 mm gapping in 10° of knee flexion and with the increase in gapping during the increase of flexion up to 2 mm. The small modification in component placement is possible due to the precise three‐dimensional planning and execution of the cuts restoring the natural ligament tension. Thus, the varus‐aligned knees prior to surgery remained in varus after UKA. Alignment studies showed that 49% of the normal population presents a natural varus alignment of 3° and more [5]. Other studies reported about a varus alignment of 3° in 29.2% of the non‐arthritic population [20]. Both studies indicated that a significant number of patients are not neutrally aligned, and these patients should not be corrected into neutral. Keeping the varus‐aligned knees in varus helps prevent overstuffing of the medial compartment and overloading of the lateral compartment, both of which may increase the risk of failure and be associated with inferior long‐term outcomes [32]. The 2.7° correction in the current study is primarily due to the restoration of cartilage thickness in the medial femorotibial compartment.

Assessing previously performed biomechanical studies is challenging because femorotibial loading and strain assessment were generally based on mechanical alignment. Modern alignment philosophies follow the patient's phenotype, making it difficult to generalise alignment and loading aspects due to variation in the kinematics [27, 30]. Some authors have demonstrated that preserving a patient's alignment reduces loading and may provide a greater likelihood of survival [10, 18]. These goals are achievable when novel technology improves the accuracy and reproducibility of component placement by respecting ligament tension during surgery. Cadaver studies have shown increased bone strain after UKA compared to the native knee despite the preservation of the three‐dimensional knee stability [4, 28]. Differences in strain may occur due to small changes in the lower limb alignment in both flexion and extension.

Overstuffing of the medial compartment may be expected when natural varus alignment is corrected to neutral during surgery, causing unphysiological tension in the medial collateral ligament and potential impingement around the patellofemoral joint. Compared to balanced UKA, overstuffing the medial compartment increases valgus alignment throughout the range of motion and distal translation of the tibia from maximum extension to 45° of flexion [8]. However, predicting the resultant force in the cruciate and collateral ligaments of the human knee is difficult due to individual variation in knee ligaments' viscoelasticity. Restoration of the natural femorotibial alignment is also important when considering the patellofemoral compartment. It has been shown that under‐correction of the pre‐arthritic alignment following UKA will cause patellofemoral incongruency [29].

The current study did not show a significant difference between the planned and final femoral and tibial implant position, except for the posterior slope of the tibial component. The posterior tibial slope was increased by 0.7° compared to the pre‐surgery plan, and its clinical relevance is questionable. Finite Element Analysis studies showed no changes in contact stress when the posterior tibial slope is within a range of 2° [21]. Preserving the posterior slope is crucial to avoid overstuffing the femorotibial compartment in flexion, which may restrict knee flexion. The natural posterior slope is 6.4° ± 3.6° for the medial tibial plateau and 5.6° ± 3.4° for the lateral plateau according to the analysis of 234 CT scans [36]. The natural posterior slope of the patients was respected up to 7°. However, when the posterior slope of the tibial component exceeds 7°, no changes in contact pressure were observed in the polyethylene insert at the medial compartment, but an increase in contact pressure occurred in the lateral compartment [36]. The increase in slope increases strain in the anterior cruciate ligament, causes a lateral shift of the patella in the trochlea during the early stage of knee flexion, increases strain in the MCL and decreases strain in the PCL [14, 34]. An analysis of the posterior tibial slope's effect on gait using a finite element model reported an increase in contact pressure at the medial compartment during the first peak of the ground reaction force, followed by a decrease during the second peak [36].

Studies have shown that robot‐assisted knee arthroplasty surgery can improve the accuracy of implant positioning and alignment compared to conventional techniques [1, 13, 24, 29]. The precise three‐dimensional placement of the components allows to restore bony contour of the medial femoral condyle and medial tibial plateau as closely as possible [22]. Improved accuracy may result in better functional outcomes, faster recovery, and reduced risk of complications such as implant loosening or premature wear [7, 30].

The Australian prosthetic registry (Orthopaedic Association National Joint Replacement Registry) demonstrated lower revision rates at 3 years of follow‐up after medial R‐mUKA compared with conventional techniques [31]. Another study reported a higher survival rate at 10 years and higher patient satisfaction after R‐mUKA [3]. At the 10‐year follow‐up, their survivorship rate was 91.7% (95% confidence interval [CI], 88.8%–94.6%). Of all revisions (29 cases), 26 UKAs were revised to total knee arthroplasty. Unexplained pain and aseptic loosening were the most common modes of failure, accounting for 38% and 35% of revisions, respectively. However, 91% of the patients were either satisfied or very satisfied with their overall knee function after UKA, which is higher than after TKA. Promising early results with a follow‐up of at least 10 years were shown by others [38]. In the study with 196 knees and a 79.4% follow‐up rate, seven R‐mUKA underwent revision, resulting in a survivorship rate of 96.4% (CI 94.6%–99.2%). Causes of revision included aseptic loosening (two cases), infection (one case), post‐traumatic (one case) and unexplained pain (three cases). The mean FJS‐12 and satisfaction were 82.2 (SD 23.9) and 4.4 (SD 0.9), respectively. Male subjects had a higher probability of attaining a ‘forgotten joint’ (*p* ≤ 0.001) and high satisfaction (equal to 5, *p* ≤ 0.05) compared to females. Other studies also reported the gender difference in revision rates and clinical and functional outcomes after UKA [5, 29]. To date, the causes of gender differences remain unclear in the literature, thus being an observational finding in the main study databases. The underlying pathogenesis leading to more precocious failure of prosthetic implantation in females than in males may be due to anatomical differences and a greater risk of overhanging prosthetic components [8, 12]. No gender differences were observed in the current study regarding the accuracy of implant placement or lower limb alignment.

Control and optimisation of surgical variables, such as implant positioning in the three‐dimensional planes, alignment of the lower limb throughout the full range of motion, soft tissue balancing, and maintenance of the level and orientation of the joint line, can reduce the number of outliers [4, 11, 19, 20]. Our results, although without clinical and functional evidence, align with those of other authors, as highlighted in several reviews and meta‐analyses. Other meta‐analyses have compared the results of conventional and robotic unicondylar knee arthroplasty [9, 12, 15]. Some authors found that robotic‐assisted surgery achieves lower limb alignment within 3° of varus and valgus alignment, which is a measure of good knee alignment, more effective than conventional surgery (MD = 0.86, 95% CI = 0.16–1.56). The accuracy was even higher in the current study, showing that component placement may depend on the surgeon's experience even when using robotic technology. The authors concluded that robotic surgery may have some advantages over conventional surgery in terms of knee alignment and function. However, no significant difference between the two methods in terms of other outcomes, such as pain, range of motion, health status and joint awareness, has been found so far [19, 35]. An image‐based system was used in the current study and may allow higher accuracy in implant positioning in comparison to non‐image‐based systems [17].

## CONCLUSION

The study underlined the accuracy in component position and the restoration of lower limb alignment when using R‐mUKA and ligament balancing, respecting the joint line orientation in both the coronal and sagittal planes. It allows optimal component position even for off‐the‐shelf implants respecting the patient's specific anatomy.

## AUTHOR CONTRIBUTION

Roland Becker and Mikhail Salzmann have designed the study. Emanuele Diquattro, Jonathan Lettner, and Marco Adriani have collected and analysed the data. Emanuele Diquattro, Robert Prill, and Roland Becker have written the manuscript. All authors reviewed and revised the manuscript.

## CONFLICT OF INTEREST STATEMENT

The authors declare no conflict of interest.

## ETHICS STATEMENT

The study was approved by the ethical committee of the state of Brandenburg (AS 113[bB]/2016). Brandenburg, 29/08/2024. All patients gave written consent.
